# Changes in the Protein Secondary Structure on the Surface of Silica Nanoparticles with Different Sizes

**DOI:** 10.1021/acs.langmuir.5c01606

**Published:** 2025-06-03

**Authors:** Naoya Sakaguchi, Atsuto Onoda, Kyoko Omata, Masakazu Umezawa

**Affiliations:** 1 Department of Materials Science and Technology, Faculty of Advanced Engineering, 26413 Tokyo University of Science, 6-3-1 Niijuku, Katsushika, Tokyo 125-8585, Japan; 2 Department of Toxicology and Health Science, Faculty of Pharmaceutical Sciences, 57952 Sanyo-Onoda City University, 1-1-1 University Street, Sanyo-Onoda City, Yamaguchi 756-0884, Japan; 3 Department of Medical and Robotic Engineering Design, Faculty of Advanced Engineering, 26413 Tokyo University of Science, 6-3-1 Niijuku, Katsushika, Tokyo 125-8585, Japan

## Abstract

Nanoparticles (NPs) are highly promising for medical applications; however, their toxicity is a limiting factor. Understanding the interactions between NPs and proteins is crucial for mitigating toxicity concerns and advancing the safe use of NPs in the biomedical field. Important factors governing NPs–protein interactions include the size (curvature), surface charge, and surface state of NPs as well as coexisting ions in solvents. In this study, we focused on the effect of the NP size (curvature) on the protein secondary structure using silica NPs (SiNPs) with diameters of 10 nm, 100 nm, 1 μm, and 10 μm. The secondary structure of bovine serum albumin (BSA) that interacted with SiNPs was analyzed via thioflavin T (ThT) fluorescence, Fourier transform infrared spectroscopy (FT-IR), and circular dichroism (CD). Furthermore, the stirring time was varied to 1, 24, and 48 h, and the effect of the incubation time was investigated. ThT measurements showed that the β-sheet ratio of BSA was the highest when incubated with SiNPs of 10 nm diameter for 1 h. This result can be attributed to the characteristics of small SiNPs such as high curvature and large surface area per mass, facilitating more extensive interactions with BSA. Interestingly, the dependence of the ThT fluorescence intensity on the NP diameter did not show a linear pattern. This is potentially caused by a complex interplay of factors including changes in the curvature and the total surface area of SiNPs. Notably, ultrasmall SiNPs exhibited the potential to induce an abnormal protein conformation. The relationship between the SiNP size and protein secondary structure change presented in this study sheds light on critical factors for the safe and effective application of NPs in future biomedical applications.

## Graphical abstract

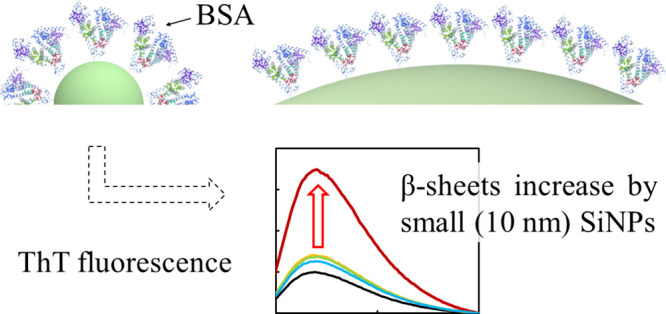

## Introduction

Nanoparticles (NPs) are defined as solid colloidal particles with sizes ranging from 10 to 1000 nm.^1^ Due to their large specific surface area and ability to penetrate deep into the body, NPs are highly promising for various biomedical applications such as bioimaging, drug delivery systems, hyperthermia, photoablation therapy, and biosensors.^2^ However, when NPs are administered in vivo, interactions with proteins and other biomolecules occur, increasing concerns that NPs may cause biotoxicity. For example, CeF_3_ NPs, which are promising for bioimaging, may promote the formation of the β-sheet structure of amyloid β-peptides and increase the risk of developing Alzheimer’s disease.^3^ Exposure of mouse mothers to carbon black NPs induced perivascular endoplasmic reticulum stress due to the accumulation of misfolded proteins in the developing offspring brain.^4^ A previous study by Khanal et al.^5^ using IR spectroscopy revealed alterations of the secondary structure of fibronectin by nanodiamonds, concluding that the behavior of individual NPs in cells must be explored at the nano- and molecular level to establish the safety of nanomaterials. Thus, NPs entering the body may lead to abnormal conformations, especially of proteins, resulting in biotoxicity. Therefore, establishing the safety of NPs is an essential topic for the biological application of NPs, and clarifying the effects of NPs on the secondary structure of proteins is highly important.

Among inorganic NPs with potential biomedical applications, silica NPs (SiNPs) are some of the most commonly used substrates. SiNPs are composed of silicon dioxide (SiO_2_) and are promising for biomedical applications such as bioimaging and drug delivery systems due to their biocompatibility and nontoxicity.^6^ In addition, because they contain oxygen atoms with high electronegativity, their surface polarity and hydrophilicity are relatively higher than those of other inorganic NPs.

When the biotoxicity of NPs is considered, it is important to consider the secondary conformational changes of proteins that occur at the NP surface. For example, the presence of hydrophobic NPs in a system may promote the aggregation of proteins on the NP surface through hydrophobic interactions. In the case of charged NPs, electrostatic interactions between NPs and the amino acid residues constituting the protein, which are also charged, also affect the aggregation tendency of the protein. Roach et al.^7^ examined the changes in the secondary structure of bovine serum albumin (BSA) and fibrinogen (Fg) on the surface of SiNPs with different radii (7.5–81.8 nm) via FT-IR. They revealed that the percentage of disordered structures increases with increasing SiNPs for BSA, while Fg loses order when adsorbed on NPs with high surface curvature. The adsorption of chicken egg lysozyme on SiNPs of various diameters was studied using SiNPs with various diameters (4, 20, and 100 nm).^8^ Circular dichroism (CD) showed that the α-helix content of chicken egg lysozyme increased when the curvature of SiNPs increased, while their diameters decreased. The composition and structure of the protein corona formed on SiNPs (diameter: 8.3, 33.0, and 78.0 nm) in soluble yeast protein extracts were also investigated.^9^ Adsorption isotherms showed that the amount of adsorbed protein varied greatly with the NP size, with larger NPs adsorbing more protein per unit surface. The composition of the protein corona was validated using an extensive label-free proteomic approach combined with statistical and regression analyses, revealing that the composition of proteins adsorbed on the NPs was almost identical regardless of the NP size.

The purpose of this study was to determine the effect of NPs of different sizes on the protein secondary structure. To the best of our knowledge, no study examining such a wide range of NP diameters (10 nm–10 μm) has been reported. To achieve our purpose, we used SiNPs, which are commonly used as substrates, and albumin, which is the most abundant protein in serum and cerebrospinal fluid. Furthermore, the changes in the secondary structure of BSA on the SiNP surface were analyzed via ThT fluorescence, FT-IR, and CD measurements.

## Experimental Section

### Materials

BSA and deuterium oxide (D_2_O) were purchased from Sigma-Aldrich (St Louis, MO, USA). All SiNPs (sicastar; 10 nm, 100 nm, 1 μm, and 10 μm) with a plain surface were purchased from micromod Partikeltechnologie GmbH (Rostock, Germany). ThT (Basic Yellow 1) was purchased from Tokyo Chemical Industry Co. (Tokyo, Japan). PBS was purchased from Gibco Life Technologies (Grand Island, NY, USA).

### Dynamic Light Scattering (DLS) and Zeta Potential Measurements

Each SiNP of 10 nm–10 μm in diameter was dispersed in deionized water at a concentration of 100 μg/mL. Size distribution was analyzed by dynamic light scattering (DLS) using a laser scattering particle size distribution analyzer LA-960 (Horiba, Ltd., Kyoto, Japan), and zeta potential was analyzed using a nanoparticle analyzer SZ-100 V2 (Horiba).

### ThT Fluorescence

Thioflavin T (ThT) is a fluorescent reagent that fluoresces upon adsorption to amyloid fibers such as amyloid β and α-synuclein. It is most commonly used to observe β-sheet formations.^10^ ThT (50 μM) and BSA (30 μM) were dissolved in PBS and mixed with SiNPs (diameter: 10 nm, 100 nm, 1 μm, or 10 μm) at a concentration of 0.3 mg/mL. After stirring for 1, 24, and 48 h, the samples were irradiated with an excitation light at 442 nm and fluorescence was observed at 400–600 nm by using a fluorescence spectrometer RF-5300PC (Shimadzu Co., Kyoto, Japan). Each measurement was taken five times, and the mean and standard deviation were calculated. When bound to amyloid, ThT exhibits fluorescence at 485 nm upon excitation at 442 nm; ThT consists of an *N*,*N*-dimethylaniline moiety (electron donor) and a benzothiazole moiety (electron acceptor). When excited by light irradiation, the donor–acceptor single bond undergoes intramolecular rotation around the donor–acceptor bond and rapidly relaxes to the ground state, resulting in the absence of fluorescence. In contrast, when ThT binds to the cross β-sheet structure of aggregates, this binding suppresses intramolecular rotation, resulting in fluorescence and relaxation.^11,12^

### FT-IR

Water-dispersed SiNPs were lyophilized, and the solvent was replaced with D_2_O. BSA (30 mg/mL) and SiNPs (4.5 or 9.0 mg/mL) were mixed and stirred for 1, 24, or 48 h. The obtained samples (20 μL) were placed onto the sample stage to perform liquid film FT-IR measurements. FT-IR spectra including amide bands were recorded using an FT/IR-6200 spectrometer (Shimadzu) for the mixed samples sandwiched between two CaF_2_ plate windows (spacer of 0.025 mm), and 100 scans were averaged for each FT-IR spectrum. FT-IR analysis of the amide I vibration of proteins in solution is usually performed in heavy water (D_2_O) instead of water (H_2_O)^13^ because the absorption originating from the HOH-divergent vibration of H_2_O is observed at approximately 1650 cm^–1^, which overlaps with the absorption originating from the amide I vibration of the protein, making observation difficult. Measurements were also made for systems containing only solvent and SiNPs for subtracting background from the data of each sample.

### Circular Dichroism (CD)

CD was analyzed using a JASCO J820 model, with the light source, monochromator, and sample chamber replaced with dry nitrogen gas. A quartz semimicrocell with an optical path length of 10 mm and an optical path width of 4 mm was used at 0.1 nm intervals, with three scans averaged for each CD spectrum in the range 200–250 nm. The final concentration of BSA was 20 μg/mL and that of SiNPs was 0.3 mg/mL in PBS. The measurements were taken after overnight incubation. The obtained data were smoothed by using the Savitzky–Golay method. Secondary structure percentages were determined using the BeStSel (Beta Structure Selection) web server.^14,15^

## Results and Discussion

DLS and zeta potential were measured to evaluate the physical properties of the SiNPs. From the DLS measurement results shown in Figure 1, the peak positions were found to be 16.84, 105.1, 837.1, and 13250 nm, which allowed us to determine the exact dynamic particle size of the SiNPs used in this experiment. The zeta potential of each SiNP was also measured and is shown in Table 1. It can be seen that the sliding surfaces of all SiNPs are negatively charged. This is due to surface deprotonation of the silanol groups.

**Figure 1:**
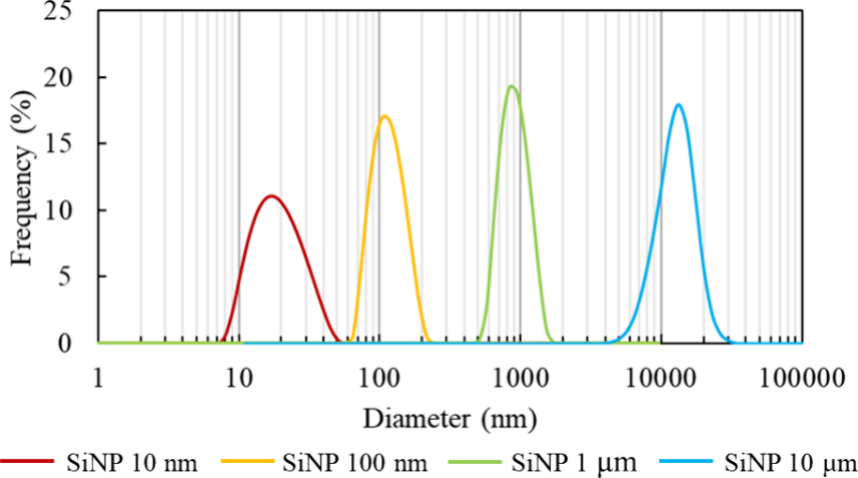
Size distribution of each SiNP.

**Table 1:** Zeta Potential of Each SiNP^a^

diameter (nm)	zeta potential (mV)
10	–55.0 ± 2.98
100	–86.7 ± 0.771
1000	–96.9 ± 2.03
10000	–65.3 ± 3.34

a Zeta potential was measured three times per sample. Data are shown as mean ± standard deviation.

The effect of SiNPs with different diameters on the number of β-sheets in BSA was investigated via ThT fluorescence measurements. ThT is commonly used to detect amyloid aggregation of amyloid β- and α-synuclein. Furthermore, it has been reported to adsorb to BSA and human serum albumin protein (HSA) for fluorescence emission, as well as for tracking β-sheet changes in serum albumin protein, which is most abundant in serum and cerebrospinal fluid.^16–25^ As shown in Figure 2A, when BSA was mixed with the smallest SiNPs (10 nm) for a stirring time of 1 h, a significant increase in the ThT fluorescence intensity was observed. A slight difference was observed when BSA was mixed with SiNPs of 100 nm–10 μm, revealing that the ThT fluorescence intensity increased in the order of decreasing SiNP diameter. The ThT fluorescence increased dramatically with increasing stirring time (∼48 h) for BSA mixed with the largest SiNPs (10 μm), whereas it decreased slightly in the other cases. These differences could be attributed to the high molecular mobility of small SiNPs resulting in more BSA encounters in a shorter time and the lower mobility of larger SiNPs that take longer time to interact with BSA. A marked increase in the ThT fluorescence intensity was observed for the 10 nm and 10 μm SiNPs; however, this was not the case for the 100 nm and 1 μm SiNPs. No linear change in the ThT fluorescence intensity was observed as a function of the SiNP diameter, suggesting that the SiNPs–protein interaction was likely caused by a complex interplay of multiple factors.

**Figure 2:**
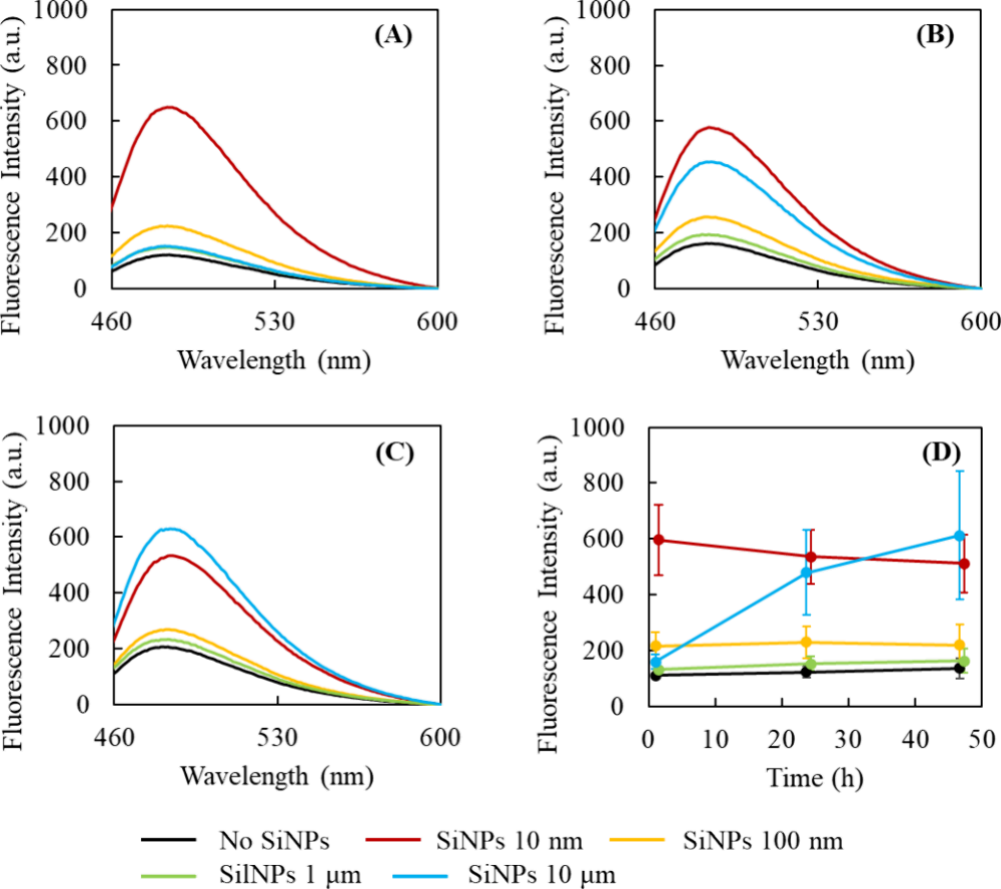
Effect of SiNPs of different diameters on the amount of β-sheets in BSA evaluated via ThT fluorescence. ThT fluorescence (excitation wavelength 442 nm) was measured on samples containing 30 μM bovine serum albumin protein with and without 0.3 mg/mL SiNPs after stirring for (A) 1, (B) 24, and (C) 48 h. (D) Changes in fluorescence intensity at 485 nm plotted against time. ThT fluorescence was measured five times per condition, and standard deviations are indicated by error bars.

BSA and SiNPs were mixed at a mass ratio of 6.6:1 in the experiments using ThT. FT-IR analyses were performed by mixing 30 mg/mL BSA with 4.5 and 9.0 mg/mL SiNPs. FT-IR is widely used to analyze the secondary structures of proteins either in solution or in the solid state.^26–29^ Protein-derived absorption in the infrared spectrum includes amide I and II vibrations. Amide I vibrations are located at approximately 1650 cm^–1^ and mainly involve C=O stretching vibrations, with minor contributions from CN stretching, CCN deformation, and NH in-plane bending. Amide I vibrations are largely independent of side chain properties. Amide II vibrations are observed at approximately 1550 cm^–1^ and are attributed to an out-of-phase combination of NH in-plane bending and CN stretching vibrations, with minor contributions from CO in-plane bending of CO and CC and NC stretching vibrations. Similar to amide I vibrations, amide II vibrations are largely unaffected by side chain vibrations; however, the correlation between the secondary structure and frequency is more complex than in the case of the amide I vibrations.^30^

The results of the Gaussian fitting of the IR absorption spectra of BSA are shown in Figure 3. The main absorption peak is observed at approximately 1650 cm^–1^, whereas the absorption of amide I and II is located at approximately 1570 cm^–1^. The amide I absorption provides more detailed information regarding each protein secondary structure, with α-helices, β-sheets, intermolecular extended chains, and β-turns.^30^

**Figure 3:**
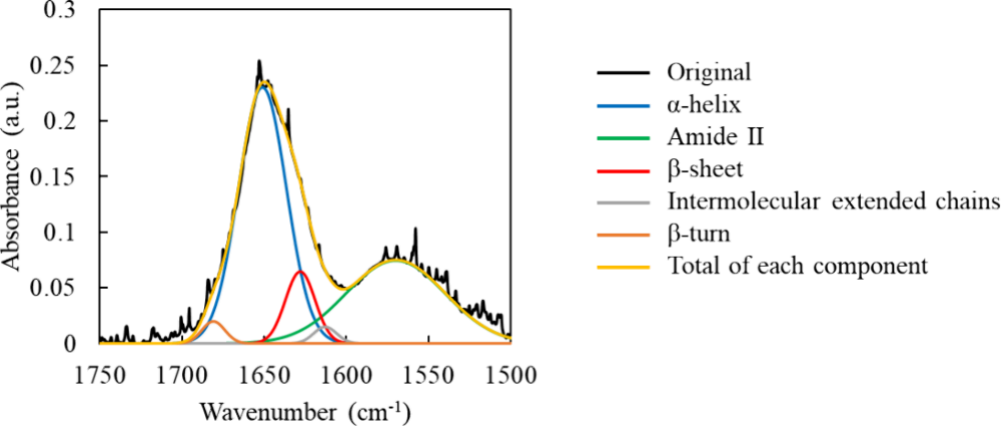
Gaussian fitting results of the infrared absorption spectrum of BSA (without SiNP, stirring time 1 h). Absorption peaks are assigned as follows: α-helix 1651 cm^–1^, amide II 1570 cm^–1^, β-sheet 1628 cm^–1^, intermolecular extended chains 1613 cm^–1^, and β-turn 1681 cm^–1^.^30^

The ratios of α-helices to β-sheets in the total absorption of the amide I-derived BSA were calculated from the results of Gaussian fitting of the FT-IR data, as shown in Figure 4. The amount of α-helix in BSA decreased and the amount of β-sheet increased after 24 and 48 h of stirring in the presence of 10 μm SiNPs (9.0 mg/mL). Furthermore, when stirred with SiNPs 10 nm (4.5 mg/mL) for 1 and 24 h, the amount of β-sheet increased slightly compared to when no SiNPs were added; however, after 48 h of stirring, it decreased slightly. The significance of this small change is not clear and it is not as pronounced as that observed in the ThT fluorescence measurement. These results suggest that FT-IR measurements can provide a crude representation of the protein secondary structure; however, they are not as sensitive as the ThT fluorescence measurements.

**Figure 4:**
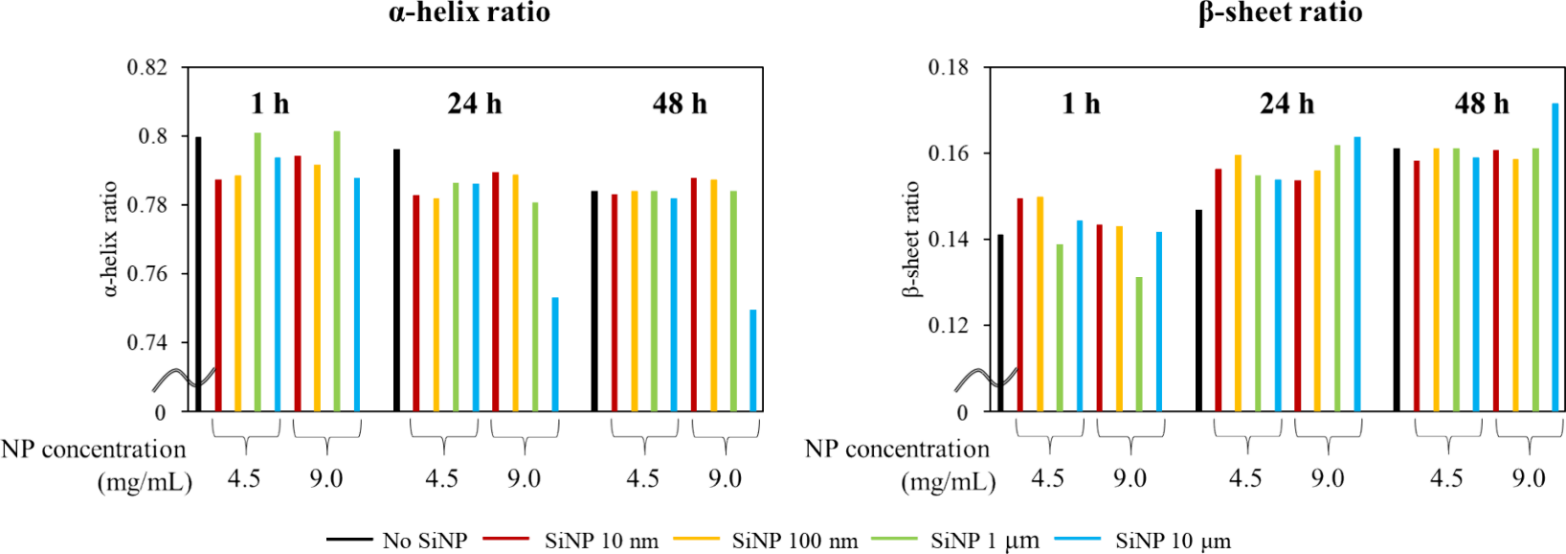
Ratios of α-helix and β-sheet in total absorbance of amide I-derived BSA. Absorbances of α-helix and β-sheet are shown on the left and right, respectively, and are classified according to stirring times of 1, 24, and 48 h.

The CD spectrum of BSA in the presence of 10 nm–10 μm diameter SiNPs is shown in Figure 5. The negative maxima were found at 208 and 222 nm, which were attributed to the α-helix structure.^31–36^ The negative maxima at 208 and 222 nm were the largest for the no SiNP system, while they were the smallest for the 10 μm SiNPs. This suggested that BSA had the largest α-helix in the no SiNP system and the smallest α-helix in the SiNP 10 μm system. However, it is known that the negative maximum derived from the β-sheet is around 218 nm^37,38^ and could be hidden under the spectrum derived from the α-helix. This suggests that it is generally inaccurate to determine the α-helix abundance from the absolute amounts of CD (mdeg) at 208 and 222 nm. Therefore, we further analyzed the CD spectra using BeStSel, which is sensitive to β-sheet-derived peaks.^14,15^ The results are presented in Table 2. It shows that the α-helical structure of BSA was disrupted by SiNPs, and the effect was greater as the diameter of the SiNPs increases. The other secondary structure that increased most with the decrease in the α-helix structure was found to be the parallel β-sheet; unlike in the case of 1 μm SiNPs, the ratio of antiparallel β-sheets and other secondary structures was also higher.

**Figure 5:**
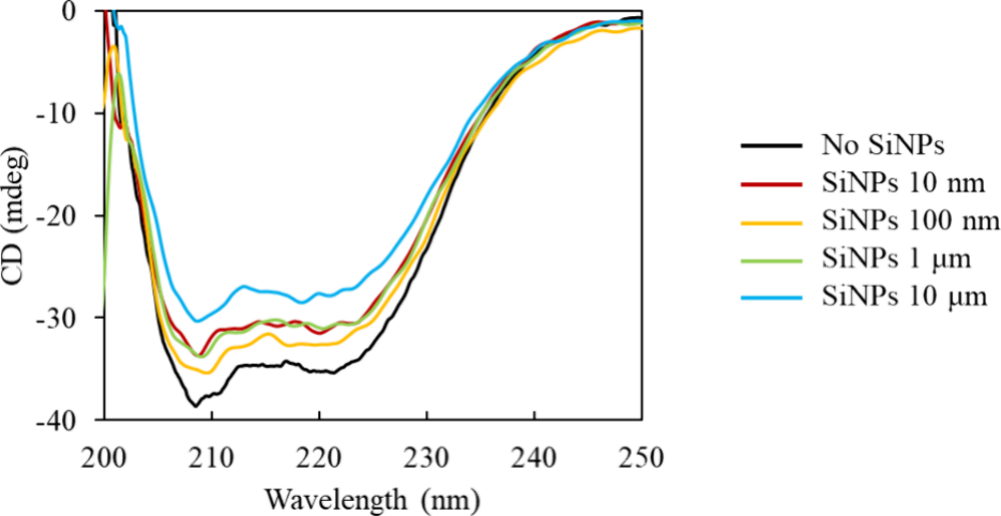
CD spectra of BSA in the presence of SiNPs with different diameters (10 nm–10 μm).

**Table 2:** Percentage of Each Protein Secondary Structure Determined by BeStSel^14,15^ from the CD Spectra of BSA in the Presence of SiNPs of Different Diameter (10 nm–10 μm)

	no SiNPs	SiNPs 10 nm	SiNPs 100 nm	SiNPs 1 μm	SiNPs 10 μm
helix	86.7	83.3	76.3	74.2	72.2
antiparallel	0	0	0	8.9	0
parallel	13.3	11.8	23.7	5.2	25.9
turn	0	0	0	0	0
others	0	4.9	0	11.7	1.9

The surface area per volume of particles is inversely proportional to their size (Figure 6A). The greater the total surface area of the particles, the more particles the protein can contact and thus the more likely it is that the secondary conformational change of the protein by the particles will occur.

**Figure 6:**
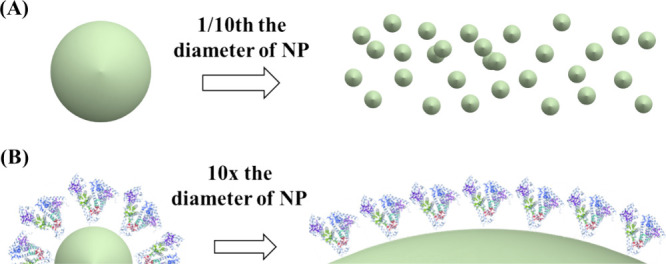
Potential mechanisms of the NP size effect on the protein secondary structure. (A) Decreasing the diameter of NPs increases the total surface area of NPs. (B) Effect of NP curvature on the protein secondary structure when the NP diameter is increased by a factor of 10.

Figure 6B shows the changes in the size of NPs and proteins as the diameter of NPs is increased by a factor of 10. As the particles become larger, their surface approximates a flat surface, and the positional relationship of the proteins on this surface changes. This change in the positional relationship of the proteins is thought to affect the ease with which the secondary structure can be formed between the individual proteins.

## Conclusions

The effect of SiNPs sizes on the secondary structure of BSA was investigated using SiNPs with diameters of 10 nm, 100 nm, 1 μm, and 10 μm, revealing no linear changes in the ThT fluorescence intensity as a function of the NP diameter. This phenomenon was attributed to a complex interaction of several factors, including changes in the curvature of SiNPs and the total surface area of SiNPs per unit volume. ThT fluorescence measurements showed a significant increase in the number of β-sheets in the presence of 10 nm SiNPs. Such a result suggests that the formation of a β-sheet structure on the particle surface was promoted by the increase in the total surface area of the particles as well as the increase in the contact area between the protein and the particles as the particles became smaller. FT-IR measurements revealed a decrease in the α-helix ratio and an increase in the β-sheet ratio after prolonged stirring in the presence of 10 μm SiNPs, suggesting that the interaction may have taken longer due to the lower mobility of the larger particles. The CD spectra show that the α-helical structure of BSA is disrupted by the SiNPs, resulting in more parallel β-sheets, and that the effect is greater as the diameter of the SiNPs increases. SiNPs are promising for potential biological applications; however, caution should be exercised when the diameter of SiNPs is extremely small as their high curvature and high specific surface area can induce abnormal protein conformations.

## Supplementary Material



## Notes

Gaussian fitting results of the IR absorption spectra of BSA and IR absorbance attributed to each secondary structure of BSA for all conditions (PDF)
