# Supplementary material for: Changes in the Protein Secondary Structure on the Surface of Silica Nanoparticles with Different Sizes
Source: Langmuir. 2025 Jun 3;41(23):15143–8. doi: 10.1021/acs.langmuir.5c01606 (PMC12177945; doi:10.1021/acs.langmuir.5c01606)
Supplement: Supplementary file 1 [file la5c01606_si_001.pdf]

Supporting Information

Changes in Protein Secondary Structure on Surface of Silica Nanoparticles with Different Sizes

Naoya Sakaguchi[1)], Atsuto Onoda[2)], Kyoko Omata[1)], Masakazu Umezawa[1),3),*]

1) Department of Materials Science and Technology, Faculty of Advanced Engineering, Tokyo University of Science, Tokyo 125-8585, Japan

2) Department of Toxicology and Health Science, Faculty of Pharmaceutical Sciences, Sanyo-Onoda City University, 1-1-1 University Street, Sanyo-Onoda city, Yamaguchi 756-0884, Japan

3) Department of Medical and Robotic Engineering Design, Faculty of Advanced Engineering, Tokyo University of Science, Tokyo 125-8585, Japan

*Corresponding Author: E-mail, masa-ume@rs.tus.ac.jp

The results of the Gaussian fitting for all conditions of the FT-IR measurements are shown in Figures S1 ~ 3. The height of the Gaussian derived from each protein secondary structure obtained for the Gaussian fitting is shown in Table S1 ~ 3.

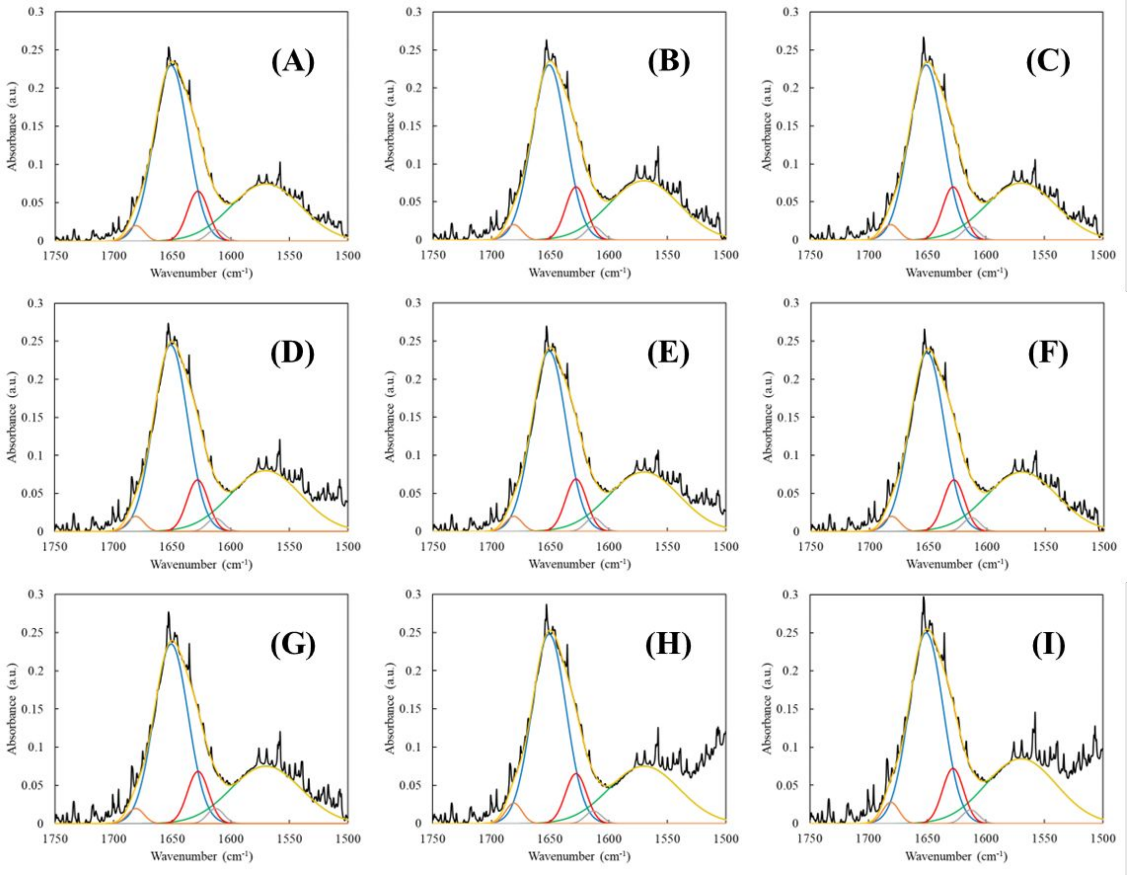

Figure S1. Gaussian fitting results of the IR absorption spectra of BSA incubated with SiNP for 1 h. (A) BSA alone, (B) BSA + SiNP 10 nm (4.5 mg/mL), (C) BSA + SiNP 100 nm (4.5 mg/mL), (D) BSA + SiNP 1 µm (4.5 mg/mL), (E) BSA + SiNP 10 µm (4.5 mg/mL), (F) BSA + SiNP 10 nm (9.0 mg/mL), (G) BSA + SiNP 100 nm (9.0 mg/mL), (H) BSA + SiNP 1 µm (9.0 mg/mL), (I) BSA + SiNP 10 µm (9.0 mg/mL).

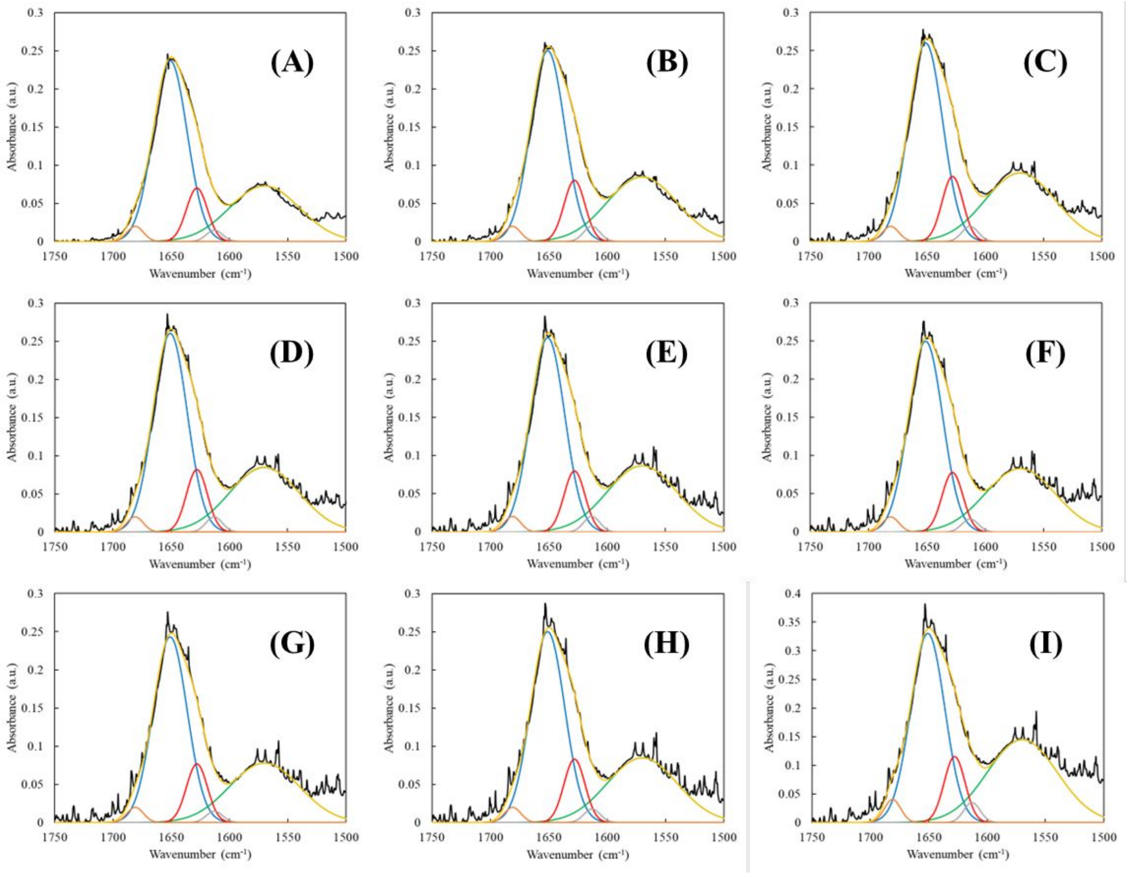

Figure S2. Gaussian fitting results of the IR absorption spectra of BSA incubated with SiNP for 24 h. (A) BSA alone, (B) BSA + SiNP 10 nm (4.5 mg/mL), (C) BSA + SiNP 100 nm (4.5 mg/mL), (D) BSA + SiNP 1 μm (4.5 mg/mL), (E) BSA + SiNP 10 μm (4.5 mg/mL), (F) BSA + SiNP 10 nm (9.0 mg/mL), (G) BSA + SiNP 100 nm (9.0 mg/mL), (H) BSA + SiNP 1 μm (9.0 mg/mL), (I) BSA + SiNP 10 μm (9.0 mg/mL).

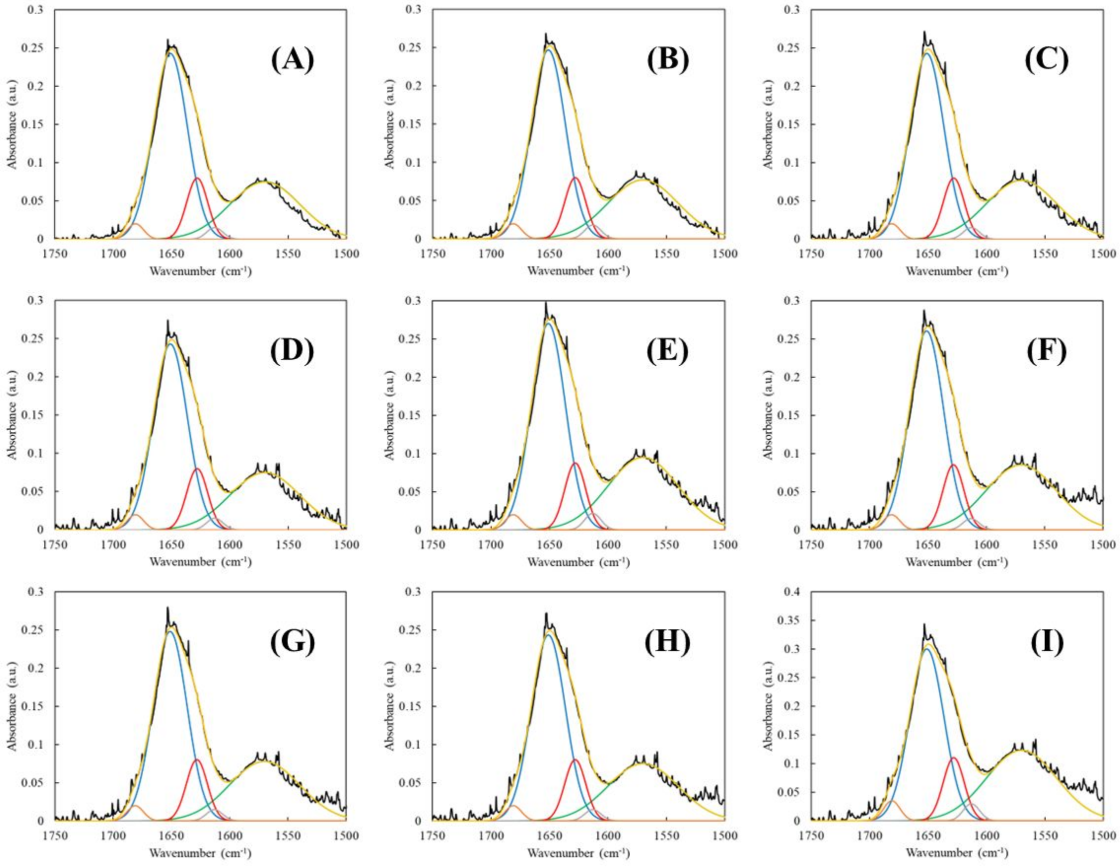

Figure S3. Gaussian fitting results of the IR absorption spectra of BSA incubated with SiNP for 48 h. (A) BSA alone, (B) BSA + SiNP 10 nm (4.5 mg/mL), (C) BSA + SiNP 100 nm (4.5 mg/mL), (D) BSA + SiNP 1 μm (4.5 mg/mL), (E) BSA + SiNP 10 μm (4.5 mg/mL), (F) BSA + SiNP 10 nm (9.0 mg/mL), (G) BSA + SiNP 100 nm (9.0 mg/mL), (H) BSA + SiNP 1 μm (9.0 mg/mL), (I) BSA + SiNP 10 μm (9.0 mg/mL).

Table S1. IR absorbance attributed to each secondary structure of BSA incubated with SiNP for 1 h.

	No NP	NP 10 nm	NP 100 nm	NP 1 μm	NP 10 μm	NP 10 nm	NP 100 nm	NP 1 μm	NP 10 μm
		4.5 mg/mL				9.0 mg/mL			
α-helix	0.23	0.23	0.23	0.245	0.237	0.235	0.235	0.248	0.25
Amide II	0.075	0.078	0.075	0.08	0.078	0.078	0.075	0.075	0.085
β-sheet	0.065	0.07	0.07	0.068	0.069	0.068	0.068	0.065	0.072
Intermolecular extended chains	0.015	0.018	0.017	0.018	0.018	0.018	0.02	0.016	0.018
β-turn	0.02	0.02	0.02	0.02	0.02	0.02	0.02	0.02	0.027
Total	0.405	0.416	0.412	0.431	0.422	0.419	0.418	0.431	0.453

Table S2. IR absorbance attributed to each secondary structure of BSA incubated with SiNP for 24 h.

	No NP	NP 10 nm	NP 100 nm	NP 1 μm	NP 10 μm	NP 10 nm	NP 100 nm	NP 1 μm	NP 10 μm
		4.5 mg/mL				9.0 mg/mL			
α-helix	0.237	0.25	0.26	0.26	0.255	0.25	0.243	0.25	0.33
Amide II	0.073	0.085	0.09	0.085	0.087	0.083	0.078	0.085	0.145
β-sheet	0.07	0.08	0.085	0.082	0.08	0.078	0.077	0.083	0.115
Intermolecular extended chains	0.015	0.02	0.02	0.02	0.02	0.017	0.015	0.018	0.035
β-turn	0.02	0.02	0.02	0.02	0.02	0.02	0.02	0.02	0.04
Total	0.415	0.455	0.475	0.467	0.462	0.448	0.433	0.456	0.665

Table S3. IR absorbance attributed to each secondary structure of BSA incubated with SiNP for 48 h.

	No NP	NP 10 nm	NP 100 nm	NP 1 μm	NP 10 μm	NP 10 nm	NP 100 nm	NP 1 μm	NP 10 μm
		4.5 mg/mL				9.0 mg/mL			
α-helix	0.243	0.247	0.243	0.243	0.27	0.26	0.248	0.243	0.3
Amide II	0.075	0.077	0.077	0.075	0.095	0.085	0.078	0.075	0.123
β-sheet	0.08	0.08	0.08	0.08	0.088	0.085	0.08	0.08	0.11
Intermolecular extended chains	0.015	0.018	0.015	0.015	0.022	0.015	0.015	0.015	0.03
β-turn	0.02	0.02	0.02	0.02	0.02	0.02	0.02	0.02	0.035
Total	0.433	0.442	0.435	0.433	0.495	0.465	0.441	0.433	0.598
